# Release of Inflammatory Mediators by Human Adipose Tissue Is Enhanced in Obesity and Primarily by the Nonfat Cells: A Review

**DOI:** 10.1155/2010/513948

**Published:** 2010-05-23

**Authors:** John N. Fain

**Affiliations:** Department of Molecular Sciences, University of Tennessee Health Science Center, Memphis, TN 38163, USA

## Abstract

This paper considers the role of putative adipokines that might be involved in the enhanced inflammatory response of human adipose tissue seen in obesity. Inflammatory adipokines [IL-6, IL-10, ACE, TGF*β*1, TNF*α*, IL-1*β*, PAI-1, and IL-8] plus one anti-inflammatory [IL-10] adipokine were identified whose circulating levels as well as in vitro release by fat are enhanced in obesity and are primarily released by the nonfat cells of human adipose tissue. In contrast, the circulating levels of leptin and FABP-4 are also enhanced in obesity and they are primarily released by fat cells of human adipose tissue. The relative expression of adipokines and other proteins in human omental as compared to subcutaneous adipose tissue as well as their expression in the nonfat as compared to the fat cells of human omental adipose tissue is also reviewed. The conclusion is that the release of many inflammatory adipokines by adipose tissue is enhanced in obese humans.

## 1. Introduction

There is increasing evidence that obesity in humans is associated with low-level inflammation [1–6] that is often accompanied by hypertension and type 2 diabetes. Currently it is thought that the increase in visceral omental rather than abdominal subcutaneous adipose tissue best correlates with measures of insulin resistance [7] and cardiovascular disease [8–10]. However, the amount of visceral fat has an allometric relationship with total body fat content [11] which means that the increases in visceral fat mass seen in obesity reflect the initial ratio of visceral fat to total fat mass as well as the changes in total fat mass change. Thus during weight loss or gain there are concurrent changes in the amount of both subcutaneous and visceral fat.

The distribution of fat between premenopausal men and women is different with women having generalized lipid deposition as contrasted to men who tend to accumulate fat in the abdominal region resulting in a socalled “beer belly”. There are also sex differences in the ratio of visceral to abdominal subcutaneous fat mass between men and women [4]. The visceral fat mass of the women was approximately 50% of the abdominal subcutaneous fat mass while for the men it was 98% [4]. 

The measurement of abdominal subcutaneous and visceral fat mass can be done using either a computed tomography (CT) or MRI scan. Measurement of total body fat requires either a DXA scan or a bioelectrical impedance scale. In contrast, waist circumference is simply measured and provides as good if not better measure of the health risks of obesity than the more complex procedures [12, 13]. However, the use of BMI has the advantage of comparing men and women on the same scale since it is an index of weight corrected for height. 

This review will primarily discuss studies on the effects of obesity on circulating adipokines, the relative release of adipokines by the fat cells versus the nonfat cells of human adipose tissue, the effects of obesity on adipokine release by explants of human visceral omental adipose tissue, and the differences in gene expression between visceral and subcutaneous fat. The term adipokine, as used in this review, means any protein released by adipose tissue without regard to whether it is released by the fat or the other cells (nonfat cells) found in human adipose tissue.

## 2. Effects of Obesity on Circulating Levels of Adipokines

At least 24 adipokines have been reported whose circulating levels are elevated in obese humans (Table 1). Some of these putative adipokines such as CRP, haptoglobin, and amyloid A are actually acute phase proteins primarily released by the liver in response to the mild inflammatory response seen in human obesity. Most of the remaining 21 are inflammatory proteins such as IL-8, PAI-1, MCP-1, IL-6, IL-1Ra, TNF*α*, sTNF RII, and IL-18 but the source of the elevated circulating levels in obesity is unclear. Their elevations could result from release by tissues other than fat. In contrast, leptin levels are elevated in obesity and the current paradigm is that it is released by fat cells in adipose tissue. However, in mice it has been shown that activated T cells and other lymphocytes can also release leptin under inflammatory conditions [14, 15]. 

**Figure 1:**
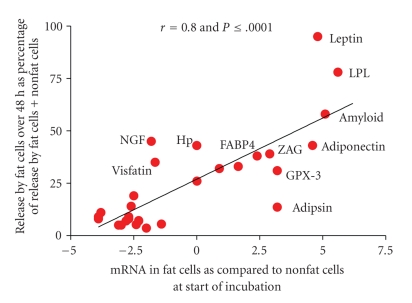
The correlation between releases of 30 adipokines over 48 hours incubation by fat cells isolated from human adipose tissue as compared to gene expression of these adipokines at the start of the incubation. The release data are from Table 1 and expressed as release by fat cells as % of that by fat cells plus nonfat cells over 48 hours. The data for mRNA are derived from those shown in Table 2 except that they are plotted as the ΔCp for the difference between mRNA in fat cells and nonfat cells instead of the ratios, which are derived from the ΔCp values. Data are not included for resistin, CRP and IL-18 since release by fat cells was below the sensitivity of the assays and mRNA was not measured for MIF, HGF, VEGF, and VCAM-1.

The circulating levels of zinc-*α*2-glycoprotein (ZAG) have been reported to be unaltered in obesity [17], but the level of ZAG gene expression in human adipose tissue is reduced in obesity [69, 70]. This illustrates the problem that changes in circulating levels of adipokines do not necessarily reflect changes in their release by or correlate with their mRNA levels in adipose tissue. Most of the adipokines are also cytokines and are released primarily by cells other than fat cells in human adipose tissue (Figure 1). Furthermore, circulating levels of all adipokines are also regulated by their release from other tissues as well as their degradation. For others such as interleukin 1*β* (IL-1*β*), no reports have been published indicating that IL-1*β* is elevated in the circulation of obese humans. However, IL-1*β* is an important regulator of the inflammatory response in human adipose tissue. It may well be a paracrine regulator that acts locally and never reaches the blood in mild inflammatory conditions such as obesity. The same may apply to PGE_2,_ which is the primary product of the cyclooxygenase-2 (COX-2) enzyme. 

Some of the adipokines may actually have anti-inflammatory effects and circulate at higher levels in obesity as part of a homeostatic mechanism to counteract the effects of the inflammatory mediators. Probably interleukin 10 (IL-10) is such a molecule [71] and there is some evidence that interleukin 6 (IL-6) has dual effects since it has been claimed that it enhances insulin action in muscle [72]. Interestingly there is also evidence that administration of a meal enhanced release of IL-6 by human adipose tissue perfused in situ [73]. It is as yet unclear whether IL-6 is enhancing or inhibiting insulin action but the traditional view is that IL-6 inhibits insulin action [74]. 

While 24 putative adipokines are listed in Table 1 whose circulating levels are elevated in obesity there are only two out of 37, adiponectin and glutathione peroxidase 3 (GPX-3), whose circulating levels have been reported to be lower in human obesity. The current paradigm is that circulating levels of adiponectin are reduced in obesity [25, 33, 34]. However, the finding that circulating GPX-3 is also lower [35], if confirmed, suggests that GPX-3 may also be important. GPX-3 is unique among the five known isoforms of this enzyme since it is the only one that is secreted by cells [75]. It is a selenocysteine-containing protein with antioxidant properties. The circulating levels of GPX-3 and selenium have also been reported to be lower in patients with coronary artery disease than in age-matched controls [76].

## 3. The Relative Release of Adipokines by the Nonfat versus the Fat Cells of Human Adipose Tissue

It has often been assumed that release of an adipokine by adipose tissue is due to the fat cells. This originated with the finding by Rodbell [77] that lipoprotein lipase [LPL] is localized in the fat cells of rat adipose tissue. It was in order to solve the problem of the localization of LPL that Rodbell [78] developed the collagenase procedure for separation of insulin-responsive fat cells from the nonfat cells in rat adipose tissue. However, Cleland et al. [79] found that most of the aromatase activity in human adipose tissue, that is responsible for estrogen formation from androstenedione, was localized in the nonfat cells and most of the IL-6 release by human adipose tissue was by the nonfat cells [80]. Fain et al. [62] subsequently reported on the relative release of 11 adipokines by the nonfat as compared to the fat cells of human omental and abdominal subcutaneous adipose tissue during an in vitro incubation. Leptin was found to be released exclusively by the fat cells, while TNF*α*, hepatocyte growth factor (HGF), IL-10, IL-1*β*, PGE_2_, IL-6, vascular endothelial growth factor (VEGF) and interleukin 8 (IL-8) were primarily released by the nonfat cells. 

In vitro, the relative release of adipokines by fat cells as compared to nonfat cells derived from human adipose tissue over a 48 hours incubation indicates that the highest release by fat cells was of fatty acid binding protein 4 (FABP-4) followed by IL-8 (Table 1). The high value for IL-8 release over 48 hours is primarily due to upregulation, since the rate of release over 48 hours derived from release during the first 40 minutes was only 2% of the 48 hours release value for both fat cell and nonfat cells [61]. Adipokine release was up-regulated to the same extent in both types of cells of either omental or subcutaneous fat [61]. 

The question arises as to how well in vitro release of adipokines over the first 48 hours of primary culture by human fat cells and nonfat cells reflects the in vivo situation. That cannot be determined because it takes a two-hour digestion to separate fat cell from nonfat cells and during that time there is upregulation of the mRNAs for inflammatory cytokines such as IL-8 and IL-6 [81]. However, what can be measured is the level of gene expression in the nonfat cells versus the fat cells at the start of the incubation which can be compared to release over 48 hours. These data are shown in Figure 1 for 30 of the 37 adipokines shown in Table 1. There was an excellent correlation (Pearson correlation coefficient of 0.8) between release of adipokines over 48 hours by fat cells as % of that by nonfat cells and the initial ratio of the mRNA for the adipokine in fat cells versus nonfat cells. The data also demonstrate that leptin release is exclusively by the fat cells of omental adipose tissue, which also contained 28-fold more leptin mRNA than the nonfat cells (Figure 1). Release of LPL was also primarily by the human fat cells and in agreement with the 79-fold greater amount of its mRNA found in fat cells as compared to nonfat cells. 

 Adiponectin has generally been considered to be an adipokine released exclusively by fat cells but while the ratio for mRNA expression in fat cells as compared to nonfat cells was 42-X the release of adiponectin accounted for only 40% of total release. Fain et al. [82] suggested that immature fat cells or other cells in the nonfat cell fractions of human adipose tissue also release adiponectin. Alternatively, the release could be due to adiponectin taken up by nonfat cells in vivo and then released during the 48 hours incubation. The release of amyloid A by human fat cells as % of that by nonfat cells was actually higher than that of adiponectin and its mRNA content in fat cell was 34-fold greater than that in nonfat cells. However, amyloid, like adiponectin, release appears to be about the same by nonfat as by fat cells. While leptin, LPL, amyloid A, and adiponectin are adipokines predominantly expressed in fat cells at ratios 30 to 80-fold greater than in nonfat cells (Figure 1), the question of whether there is appreciable amyloid and adiponectin synthesis by the nonfat cells of adipose tissue remains to be established. 

There are four other possible candidates for the designation of adipokines preferentially released by fat cells, since the ratios of their mRNAs in fat cells to nonfat cells ranged from 5 for FABP-4, 8 for ZAG, and 9 for adipsin/complement D as well as GPX3. However, release by fat cells accounted for less than half of their total release.

## 4. Relative Expression of 100 Genes in Fat Cells versus the Nonfat Cells of Human Omental Adipose Tissue

Table 2 shows the relative gene expression in fat cells versus nonfat cells of 100 proteins, as determined by qRTPCR [83]. These proteins were chosen because they are important in inflammation or obesity, regulatory proteins or proteins enriched in fat cells. 

Of the proteins whose gene expression is shown in Table 2 almost one-third (30) were significantly enriched in fat cells (shown in Bold), 29 were distributed equally (shown in italic) and 41 were significantly enriched in nonfat cells of human omental adipose tissue (shown in normal text). Thirty of these proteins are the adipokines whose release by adipose tissue was examined in the studies shown in Table 1 and Figure 1. 

Of special interest was the finding that 11*β* HSD1, UCP-2, cyclic AMP phosphodiesterase 3B, AQP7, angiotensinogen, GPX-3, the insulin receptor, and NQO1 are preferentially localized in fat cells [83]. Interestingly ZAG, TLR4, cytochrome C oxidase, Akt2, adrenomedullin, and UCP-1 were also expressed at levels 4 to 8-fold greater in fat cells than in nonfat cells [Table 2]. The higher expression of ZAG in human fat cells than in nonfat cells confirms the report by Bao et al. [84].

An elevated expression in fat cells was seen for both cytochrome C oxidase, which is a marker for mitochondria, and Akt2, which is the isoform of Akt involved in insulin-stimulated glucose uptake into fat cells [85]. The enhanced expression of the mitochondrial protein UCP-1 in visceral omental fat cells was unexpected since it is thought of as a marker for brown fat cells. However, Sacks et al. [86] found far higher expression of UCP-1 in visceral epicardial fat as compared to subcutaneous fat. The increased expression of cytochrome C oxidase in fat cells as compared to nonfat cells of omental fat suggests that fat cells are relatively enriched in mitochondria. Deveaud et al. [87] have shown that cytochrome C oxidase is enriched in visceral epididymal fat of rats as compared to subcutaneous inguinal fat. 

The circulating levels of adrenomedullin are elevated in human obesity [88, 89]. Furthermore, adrenomedullin is secreted by fat cells [90, 91] but it is unclear whether more adrenomedullin is secreted by fat cells than by the nonfat cells of human adipose tissue [88–91]. 

The proteins whose gene expression was predominantly in the nonfat cells included all the classical inflammatory proteins such as MCP-1, TGF*β*1, IL-6, IL-8, COX-2, PAI-1, IL-1*β*, IL-8, and TNF*α* (Table 2). Other putative adipokines, such as vaspin, endothelin-1, omentin/intelectin, lipocalin-2, RANTES, and visfatin were also enriched in the nonfat cells. Vaspin is an adipose tissue-derived serpin whose gene expression in human visceral fat positively correlated with obesity [92]. Circulating levels of omentin/intelectin are lower in obesity [93] but the meaning of this is unclear. 

The ratio of gene expression in fat cells to nonfat cells ranged from 0.06 to 128 (Table 2). However, if in vitro differentiated human omental adipocytes were compared to omental preadipocytes the ratios ranged from 0.001 to over a million for adiponectin [82, 83]. Clearly there is more expression of fat cell specific proteins in freshly isolated nonfat cells than in preadipocytes obtained by culturing the nonfat cells of human omental fat. This difference may be accounted for, in part, by the presence of small fat cells without enough fat to float, since isolated fat cells are operationally defined as cells containing enough lipid to float in isotonic incubation buffer. Another possibility is incomplete digestion of adipose tissue leaving some fat cells entrapped in the undigested tissue matrix. However, if this is the case these cells secrete very little leptin since its release by the nonfat cell fraction is less than 5% of that by isolated fat cells (Figure 1) and we could find no detectable fat in the nonfat cells [67]. 

One problem in comparing gene distribution between fat and nonfat cells is the possibility of preferential lysis of extremely large fat cells during the collagenase digestion of fat from extremely obese humans. The isolation of human fat cells is an art requiring particular batches of collagenase for optimal yield of responsive cells, gentle incubation conditions and an optimal ratio of collagenase to tissue [62, 67]. Fain et al. [67] calculated that there was a 23% greater loss of fat cells during digestion than of nonfat cells during the digestion of fat from extremely obese humans. The fat cells lost during digestion may well be the largest fat cells that release more inflammatory adipokines and leptin than the smaller cells. A further problem is the up-regulation of inflammatory response genes during the 2 hours required for collagenase digestion but this affects both fat cells and nonfat cells to the same extent [61] and thus has minimal effects on the ratios of mRNA expression in fat to nonfat cells.

## 5. Comparison of mRNA Expression in Isolated Omental Fat Cells versus In Vitro Differentiated Adipocytes

Many studies on the relative gene expression of proteins in fat cells have utilized adipocytes differentiated in vitro such as murine 3T3L1 cells, but far fewer studies have appeared using human cell lines. The term fat cells is operationally defined as those cells that float and are isolated by collagenase digestion of human omental adipose tissue from women undergoing bariatric surgery. Adipocytes are those fat cells derived from the adipose tissue of the same group of women that underwent differentiation in vitro in the presence of insulin, dexamethasone, a methyl xanthine, and a thiazolidinedione. 

**Figure 2:**
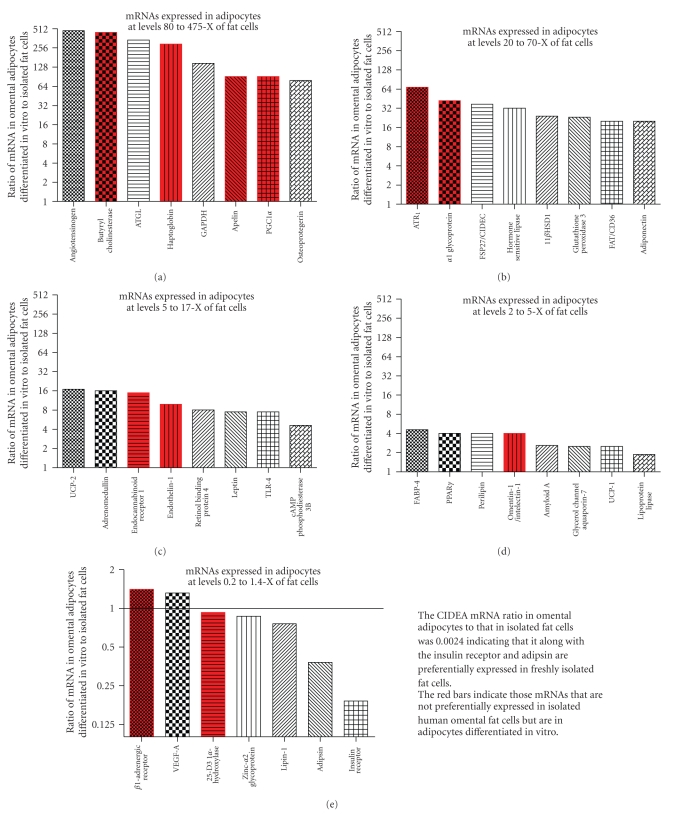
Comparison of mRNA expression in isolated omental fat cells versus in vitro differentiated adipocytes. The data are shown as the ratio of mRNA in human omental adipocytes, differentiated in vitro from the nonfat cells isolated from omental adipose tissue, to that in freshly isolated fat cells obtained by collagenase digestion of omental adipose tissue from female bariatric surgery patients. The ratios were derived from the Cp values and plotted on a log_2_ scale. Comparable amounts of total RNA were used for the mRNA analyses. The Cp values from which the ratios were determined for fat cells were calculated from the data shown in Table 2 and for in vitro differentiated adipocytes from Fain et al. [82, 83] or unpublished data. The red bars are for mRNAs whose expression in isolated fat cells was either the same or lower than in isolated nonfat cells.

In the data shown in Figure 2 the mRNA content of freshly isolated omental fat cells versus in vitro differentiated adipocytes was compared using total RNA as the recovery standard as suggested by Bustin [94] since the expression of cyclophilin A used as the recovery standard differed significantly between fat cells and in vitro differentiated adipocytes. The data indicate that many proteins are expressed at far higher levels in adipocytes than in freshly isolated fat cells. Some proteins that are expressed at higher levels in adipocytes than in fat cells are not enriched in freshly isolated fat cells as compared to nonfat cells (Figure 2). These are shown in red and are: butyryl cholinesterase, haptoglobin, apelin, PGC1*α* (peroxisome proliferator activator receptor-*γ* coactivator 1*α*), ATR_1_ (angiotensin II receptor 1), *α*l glycoprotein, endocannabinoid receptor 1, endothelin-1, and omentin/intelectin. 

Five mRNAs were found at comparable levels in adipocytes as compared to fat cells. These were the *β*1 adrenergic receptor, 25-hydroxyvitamin D3 1*α* hydroxylase, VEGF-a, ZAG, and lipin-1. Three genes were expressed at lower levels in adipocytes than in fat cells: adipsin, insulin receptor, and CIDEA. The data suggest that the one or more of the added factors required for differentiation of preadipocyes to adipocytes induce the expression of many proteins that are not induced in vivo and decrease the expression of others such as CIDEA and the insulin receptor. Clearly the use of human adipocytes differentiated in vivo from preadipocytes does not result in a pattern of gene expression comparable to that seen in intact fat from obese women.

## 6. Effect of Obesity on In Vitro Adipokine Release by Explants of Human Adipose Tissue

**Figure 3:**
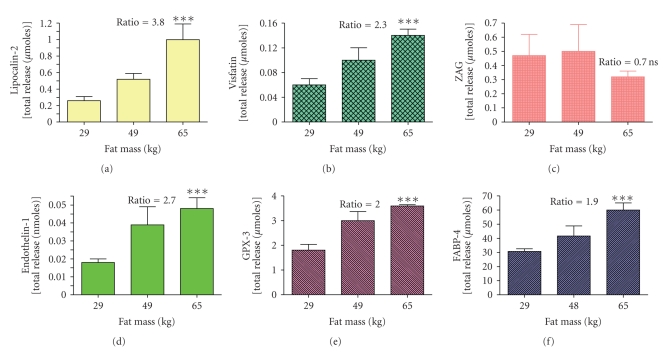
The effect of obesity on total release of 6 adipokines by explants of adipose tissue from obese women. The data are from the report by Fain et al. [42] for release of 6 adipokines by adipose tissue from 22 women divided into tertiles. The lowest tertile was composed of 7 women with total fat masses of 18 to 40 kg with a mean of 29 kg. The middle tertile was composed of fat from 8 women with total fat masses ranging from 41 to 52 kg with a mean of 49 kg. The highest tertile was fat from 7 women with fat masses ranging from 56 to 75 kg (mean of 65 kg). The ratio of total release by the highest tertile as compared to the lowest tertile is shown and all ratios were significant with a *P* < .001 except for zinc *α*2 glycoprotein [ZAG] release that was not statistically significant (*P* > .05).

Studies using freshly isolated explants preserve the cross talk between the various types of cells in fat. However, since the primary effect of obesity is to increase adipose tissue mass, it is difficult to know how to express data obtained by primary culture of human fat explants. How do you compare total release by adipose tissue from humans with 20 kg of fat as compared to those with 40 kg? In the studies shown in Figure 3 release in vitro over a 48 hours incubation of omental and subcutaneous fat from each woman per kg of fat was multiplied by the total fat content. The women were then divided by tertiles based on body fat content. 

There was enhanced release of endothelin-1, lipocalin-2, visfatin, GPX-3, and FABP-4 by the most obese women as compared to that by women in the bottom tertile (Figure 3). For ZAG we found no effect of obesity since total release was not significantly higher in women in the highest tertile but they had 124% more fat than women in the lowest tertile. Therefore there was actually decreased release per g of adipose tissue. This is in agreement with reports that gene expression of ZAG in fat is reduced in human obesity [69, 70]. There was enhanced total release of intercellular cell adhesion molecule 1 (ICAM-1), CD14, and LPL but not of osteoprotegerin, RANTES or amyloid A [42]. 

**Figure 4:**
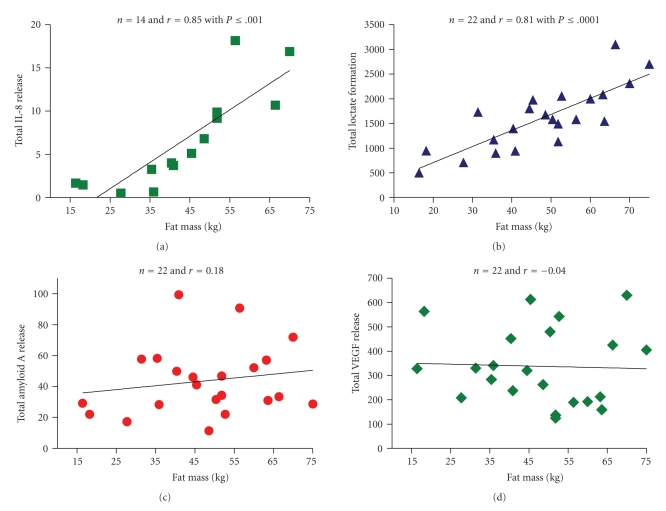
Correlation between total release of IL-8, VEGF, Amyloid A, and lactate by adipose tissue and total fat mass. The total release was calculated by averaging release over 48 hours per kg by explants of visceral omental and subcutaneous adipose tissue from 14 [IL-8] or 22 different women (lactate, amyloid A and VEGF) and multiplying by the total fat mass. Tissue samples were from the same women described by Fain et al. [42]. The Pearson correlation coefficients (*r*) are shown on the figure and the *P* value if statistically significant with a *P* < .05.

Another way to examine the effect of obesity is to correlate total release with the total fat mass of each woman. That resulted in a correlation coefficient for lactate release of 0.81 and for IL-8 release of 0.85 based on total release plotted against the fat mass of each woman (Figure 4). A positive correlation indicates that the more fat you have the greater the total amount of lactate or IL-8, if release per g of fat remains the same. In contrast, total amyloid and VEGF release did not correlate with total fat mass indicating that their release per g of tissue was less but the total release by fat remained constant. 

Data for 24 other adipokines are summarized in Table 3, along with those for lactate, amyloid A, and VEGF and IL-8 release shown in Figure 3. Adipokines that showed no correlation, that is, those whose total release actually decreased in obesity, were MCP-1, interleukin 1 receptor antagonist 1 (IL1-Ra), adipsin, osteoprotegerin, RANTES, ZAG, cathepsin S, vascular cell adhesion cell molecule 1 (VCAM-1) and NGF*β* in addition to VEGF and amyloid A. A number of inflammatory adipokines had a significant correlation between total release and total fat mass besides IL-8 and these included, IL-10, transforming growth factor *β*1 (TGF*β*1), visfatin, IL-1*β*, IL-6, CD14, endothelin-1, ICAM-1, TNF*α*, lipocalin-2, PAI-1, and angiotensin 1 converting enzyme (ACE) that are primarily released by the nonfat cells. There was also a significant correlation between total release and fat mass for FABP-4, GPX-3, and LPL. 

A problem complicating release studies by human fat is that incubation in vitro induced an inflammatory response as judged by enhanced mRNA accumulation over the 48 hours incubation for IL-8, IL-10, TGF*β*1, visfatin, IL-1*β*, IL-6, ICAM-1, TNF*α*, lipocalin-2, PAI-1 and ACE (Table 3). Interestingly, an increase in mRNA expression over 48 hours was seen for MCP-1, osteoprotegerin, and NGF*β* whose total release was not enhanced by obesity. Furthermore there was no significant change in the mRNA expression over 48 hours of CD14, endothelin-1 or ACE while there was a marked decrease in FABP-4, GPX-3, and LPL mRNA but enhanced release in obesity. These data suggest that the in vitro inflammatory response does not mimic completely the effect of obesity. 

In conclusion, adipose tissue from extremely obese women, when incubated in vitro, releases more of a host of adipokines such as IL-8, IL-10, TGF*β*1, visfatin, IL-1*β*, IL-6, ICAM-1, TNF*α*, lipocalin-2, PAI-1, and ACE than does tissue from women with a lesser amount of fat. While TNF*α* appears to be important it is one adipokine whose mRNA and release goes up transiently during in vitro incubation of adipose tissue, but unlike other members of the inflammatory cascade its release and gene expression return to near basal values by 48 hours [61, 96].

## 7. Which Cells in the Nonfat Cell Fraction Derived from Human Adipose Tissue Are Responsible for Release of Inflammatory Adipokines?

Hellman et al. [97] reported in 1963 that obesity in the obese-hyperglycemic mouse resulted in greater accumulation of mast cells in white adipose tissue. They also pointed out that the relative nitrogen content per gram of the epididymal fat pad of the obese-hyperglycemic mouse was unchanged despite the marked reduction in the number of fat cells per g of tissue. Almost 40 years later Xu et al. [98] extended this to show that the expression of genes enriched in murine macrophages such as MCP-1, TNF*α*, CD68, and F4/80 was elevated in obese mice. They also demonstrated that all of these genes were preferentially expressed in the nonfat cells of murine white fat [98]. Weisberg et al. [99] independently published similar findings and emphasized that the size of fat cells positively correlated with the percentage of macrophages in murine adipose tissue. 

Subsequently it was demonstrated that HAM56+ macrophage accumulation in visceral omental and subcutaneous fat depots of humans also positively correlated with the diameters of the fat cells in each depot. However, at any fat cell size there were more macrophages in omental than subcutaneous fat despite the fact that the average diameter of subcutaneous fat cells was 40% greater than that of omental fat cells [100]. The use of HAM56 as the macrophage marker is important since in humans CD68 [101, 102], CD14 [102], or F4-80 [102] are much less specific macrophage markers than in mice. Similar results are shown in Table 2 in that the gene expression of both CD14 and CD68 was not significantly different between the fat cells and nonfat cells isolated from human omental adipose tissue. 

The current paradigm is that obesity results in accumulation of macrophages in adipose tissue and these are primarily responsible for the release of inflammatory mediators [98–100]. A relevant question is whether macrophages are the only mononuclear phagocytes found in adipose tissue and whether they account for all of the adipokine release by nonfat cells. The potential contribution of the other nonfat cells in human adipose tissue such as the endothelial cells of the blood vessels, the smooth muscle cells and fibroblasts as well as other mononuclear phagocytes has not been carefully examined. 

Why do macrophages localize in the white adipose tissue of obese animals? Whether enhanced lysis/death of large fat cells is the primary trigger that accounts for inflammation is unknown as well as what signal results in greater macrophage accumulation in adipose tissue. One of the functions of macrophages is to aid in the clearing of dead cells. Cinti et al. [103] suggested that macrophages are localized selectively to sites of necrotic-like cell death where they appear as crown-like structures when viewed in tissue sections. They also suggested that fat cell hypertrophy per se promotes cell death resulting in macrophage accumulation and aggregation around dead cells. The current paradigm is that the larger the fat cell the more likely it is to undergo cell death. However, a consistent finding is that human visceral omental fat cells are smaller than subcutaneous fat cells from the same individual but the macrophage accumulation is greatest in omental fat so something besides fat cell size is important [104]. Furthermore, thiazolidinediones appear to selectively enhance the breakdown of large fat cells in visceral omental fat resulting in smaller more insulin-sensitive fat cells [105]. The net effect of thiazolidinediones is to preferentially enhance deposition of fat in subcutaneous adipose tissue while decreasing that in visceral fat [105].

## 8. The Relative Expression of mRNAs in Human Epicardial, Substernal, Omental, Mesenteric, and Subcutaneous Adipose Tissues

Currently it is thought that it is the increases in visceral (intraperitoneal) rather than subcutaneous (extraperitoneal) adipose tissue is linked to the enhanced risk of diabetes, hypertension and cardiovascular disease in obesity [7–10]. Exactly how visceral adipose tissue is linked to this is unclear. It could be due to greater release of inflammatory factors by visceral fat or fatty acids and adipokines released by visceral adipose tissue that are preferentially delivered to the liver through the hepatic portal system.

The visceral fat is composed of omental and to a lesser extent mesenteric adipose tissue. The search for a major biochemical difference between these two types of visceral fat and abdominal subcutaneous fat of extremely obese women has turned up some interesting differences in gene expression (Table 4). 

 The gene expression of UCP-1, omentin, and haptoglobin in subcutaneous fat was less than 10% of that in omental fat. The data on UCP-1 confirm the initial report by Esterbauer et al. [107] that UCP-1 expression in subcutaneous fat was 12% of that in omental fat. However, the amount of UCP-1 gene expression, which is related to thermogenesis, in epicardial fat of humans is at least 9-fold greater than that in omental fat [106]. Sacks et al. [86] have postulated that the epicardial fat, which is located on the outside of the heart, serves to defend the myocardium against hypothermia. 

Another protein whose gene expression was quite low (about 1%) in subcutaneous as compared to omental fat was omentin/intelectin (Table 4). It is also expressed at 3-fold higher levels in epicardial fat than in omental fat [108]. Its preferential expression in intraperitoneal adipose tissue probably reflects the fact that the blood vessels in these tissues arise from endothelial cells of the gut during development [108]. Unlike UCP-1, which is preferentially expressed in fat cells of omental fat (Table 2), omentin/intelectin is primarily found in the endothelial cells of the blood vessels [108]. 

It is unclear why haptoglobin is expressed at such low levels in subcutaneous fat but its expression is also low in mesenteric fat (Table 4). In contrast UCP-1 is found at the same level of expression in mesenteric fat as in omental fat while omentin/intelectin is found at far lower levels in mesenteric than in subcutaneous fat. As for the low level of expression of ATR_2_ in subcutaneous fat that is probably due to overexpression of ATR_1_ in subcutaneous fat. 

Comparison of mesenteric with omental fat indicates that they have more in common with each other than with subcutaneous fat. This is especially true with regard to expression of UCP-1, prostaglandin D_2_ synthase, angiotensinogen, ZAG, NF*κ*B_1_, ATR_2_, RBP-4, IL-6, and osteopontin. 

However, MCP-1, IL-1*β*, adrenomedullin, PPAR*γ*, and PAI-1 were expressed at significantly lower levels in mesenteric than in omental fat while their expression in subcutaneous fat was the same as or higher than that in omental fat. At this time these are simply lists of similarities and differences between omental and mesenteric fat indicating that they are different tissues. It is also not yet established whether the differences in mRNA expressionbetween omental and mesenteric fat are in the fat or the nonfat cells. Furthermore we know almost nothing about the physiological differences in the metabolism and adipokine release of these two kinds of intraperitoneal fat. 

There have been many studies comparing the differences in response of isolated fat cells derived from omental as compared to subcutaneous fat and pieces of adipose tissue from these depots [109, 110]. However, the data are confusing since the results have been almost as varied as the number of reports. This is especially true for PAI-1 gene expression and protein release. Some reported greater in vitro release of PAI-1 by omental than by subcutaneous fat [10], others no difference in gene expression or protein content between omental and subcutaneous [111] while yet another group reported greater release by subcutaneous than omental adipose tissue from extremely obese humans [112]. This is a common occurrence in studies comparing omental versus subcutaneous fat of humans and it is unclear why such variable results are obtained. 

The picture with regard to leptin gene expression and release is equally controversial. While some groups have reported greater expression and secretion by subcutaneous as compared to omental fat [113, 114] another group reported no difference [110] and a similar finding is in Table 4. 

TNF*α* is one adipokine that is expressed (Table 4) and released to the same extent by human omental and subcutaneous adipose tissue [96, 115]. Another inflammatory adipokine is lL-6 that is released at higher levels by omental adipose tissue than by subcutaneous adipose tissue [62, 80] but the gene expression of IL-6 was higher in freshly isolated subcutaneous adipose tissue (Table 4).

Lipolysis is reported to be greater in adipoctyes derived from subcutaneous than from visceral adipose tissue and attributed to the greater size of the subcutaneous adipocyes [116]. However, similar levels of expression for hormone sensitive lipase (HSL) and perilipin have been reported in subcutaneous as compared to omental adipose tissue (Table  4, [117, 118]). 

Giorgino et al. [109] have reviewed the evidence that fat cells isolated from omental fat are more insulin-responsive than those from subcutaneous human fat. Higher levels of insulin receptor expression have also been seen in omental as compared to subcutaneous adipose tissue [117, Table  4]. 

The visceral fat is composed of the intraperitoneal omental and mesenteric in the peritoneal cavity as well as the intrathoracic fat depots of the substernal and epicardial fat. The latter two fat depots differ in that the epicardial surrounds the heart while the substernal fat body is a separate tissue within the thoracic cavity. Gene expression in substernal can be compared to that of epicardial fat to distinguish possible differences between these two intrathoracic depots. Fain et al. [106] found that of 45 mRNAs all except five were expressed in substernal fat at levels within 0.4 to 1.6-fold of that in epicardial fat. These were haptoglobin (21-fold greater), prostaglandin D_2_ synthase (6-fold greater), nerve growth factor (5-fold greater), VEGFR/FLT1 (5-fold greater) and *α*1 glycoprotein (2-fold greater) with greater expression in epicardial as compared to substernal fat. UCP-1 is also expressed at in epicardial fat at 5-fold higher amounts than in substernal fat [86]. Of these only UCP-1 is expressed at greater levels in fat cells than in the nonfat cells of human omental adipose tissue (Table 2). These data are compatible with the hypothesis that the fat cells in epicardial fat have a unique function as a brown fat-like tissue and could be involved in thermogenesis. 

Epicardial fat has been postulated to be an inflammatory organ releasing adipokines that contributes to coronary artery disease because of the unique anatomical relationship between this fat and the coronary arteries [119]. However, when the gene expression of IL-6, IL-1*β*, PAI-1 or cyclooxygenase-2 was compared in epicardial fat of patients undergoing coronary artery bypass surgery to that in obese individuals undergoing gastric bypass surgery their expression in epicardial fat was less than 25% of that in omental fat [106]. It could be argued that this was because the bypass patients differed in other aspects, which they did, but the expression of 20 other protein ranged from 0.4 to 1.3 in omental fat to that seen in epicardial fat. In contrast, significantly higher amounts (1.6 to 2-fold greater) of the insulin receptor, ZAG, leptin, angiotensinogen and LPL were expressed in epicardial fat as compared to that in omental fat [106]. The significance of these differences between epicardial and omental fat remains unclear but do not suggest that epicardial fat is more inflamed than omental fat. 

In conclusion, the reported differences in gene expression, hormonal sensitivity, and release of adipokines by visceral as compared to subcutaneous adipose tissue have been almost as varied as the number of reports [109, 110]. Furthermore, they provide few clues that can explain the putative harmful effect of enhanced accumulation of visceral fat. The fat cells found in visceral fat are smaller than those of subcutaneous fat from obese individuals but is that due to greater breakdown of large fat cells in visceral fat? 

There are clear differences between mesenteric and omental fat but again it is unclear what they represent. Comparisons of visceral omental versus subcutaneous fat are probably influenced by the degree of obesity and this was demonstrated for PPAR*γ* where the ratio in visceral to subcutaneous was around 0.2 at a body mass index of 20 but increased to about 1.2 in individuals with a body mass index of 50 [117]. Future studies will require the development of procedures to accurately assess the gene expression and release of adipokines by the different human adipose tissue depots under more physiological and reproducible conditions. 

Recently the microRNA (miRNA) profiles of human omental and subcutaneous have been compared in humans without or with diabetes [120]. The expression of 155 miRNAs was examined and some differences were found that were said to correlate with fat cell phenotype, obesity, and glucose metabolism [120]. However, no miRNA was found exclusively in one fat depot versus the other suggesting a common developmental profile [120]. 

I conclude that the gene expression profile of omental fat clearly differs from that of subcutaneous fat for some proteins. However, none of these differences appear to explain the putative harmful effects of visceral obesity. Furthermore, there is scant agreement in the literature with respect to most proteins. This is possibly due to small sample sizes, sex differences, age differences, the extent of obesity, and the disease status of the humans from whom fat samples were obtained. For ethical reasons samples of omental and subcutaneous fat cannot be obtained from healthy donors. Most samples of human omental fat have been obtained from individuals undergoing gallstone, gynecological, or bariatric surgery. While individuals healthy enough to undergo bariatric surgery are extremely obese, the normal weight individuals always have some underlying disease process that could affect gene expression and adipokine release.

## 9. What Is the Link between TLR-4, Enlarged Fat Cells, and the Inflammatory Response Seen in Obese Humans

Recently the toll-like receptor 4 (TLR-4), that plays an important role in innate immunity through its ability to recognize bacterial lipopolysaccharides, has been postulated to play a role in the obesity-induced inflammatory response [95, 121, 122]. A loss-of-function mutation in TLR-4 prevents diet-induced obesity in mice and the development of insulin resistance [95, 121]. In macrophages and cultured adipocytes potent inducers of TLR-4 gene expression are bacterial lipopolysaccharides resulting in the release of inflammatory adipokines [123, 124]. In a monocyte/macrophage cell line (RAW 264.7) saturated, but not unsaturated fatty acids, induced the expression of COX-2 expression via TLR-4 [123]. Schaeffler et al. [122] reported that saturated fatty acids could induce the secretion of MCP-1 and other inflammatory adipokines in murine 3T3L1 adipokines through a pathway involving TLR-4.

Lin et al. [124] originally suggested that a fully intact pathway of innate immunity was present in rodent adipocytes that could be activated by bacterial lipopolysaccharides. Subsequently, functional TLR-4 has been found in human fat cells [125, 126] and the data in Table 2 indicates that in human omental fat the gene expression of TLR-4 is 5-fold greater in fat cells than in the nonfat cells. Zha et al. [127] reported that in vitro differentiated adipocytes had more TLR-4 mRNA than did preadipocytes and that TNFa secretion was induced by free fatty acids. My laboratory has similar findings in that the TLR-4 mRNA expression in human omental adipocytes differentiated in vitro was also 5-fold higher than that in preadipocytes (John N. Fain, unpublished experiments). In omental adipose tissue explants incubated for 48 hours TLR-4 gene expression was down regulated by about 70% but this was blocked in the presence of dexamethasone [128]. This may reflect a down-regulation of TLR-4 secondary to the 90 to 700-fold activation of the expression of inflammatory cytokines such as I-8, IL-6 and IL-1*β* that was markedly inhibited by dexamethasone [128].

It has been suggested that the hypertrophied fat cells seen in extreme obesity release large amounts of saturated fatty acids secondary to macrophage-induced lipolysis occurring in fat cells [129]. There is evidence in rodent adipocytes that bacterial lipopolysaccharides can stimulate lipolysis via TLR-4 [130]. However, addition of bacterial lipopolysaccharides to explants of human adipose tissue incubated for 48 hours enhanced release of IL-1*β*, IL-6, and IL-8 by 50% to 70% under conditions where there was no significant increase in lipolysis (John N. Fain, unpublished experiments). Possibly breakdown of hypertrophied fat cells could be the primary trigger for the inflammatory response via activation of TLR-4 by fatty acids in neighboring intact fat cells resulting in the release of inflammatory adipokines that cause monocyte recruitment into the adipose tissue and insulin-resistance. However, this hypothesis is probably an over-simplification since thiazolidinediones appear to enhance the breakdown of large fat cells and the accumulation of small fat cells but this is associated with enhanced insulin sensitivity [105].

It was surprising to find TLR-4, whose function has traditionally been thought of as being involved in pathogen-associated molecular recognition by immune cells, expressed at higher levels in fat cells than in nonfat cells in human fat cells. The physiological function, if any, of this enhanced expression remains to be elucidated. Another unanswered question is what is the primary trigger that results in the accumulation of activated macrophages in the adipose tissue of extremely obese humans?

## 10. Hypoxia as the Primary Trigger of the Inflammatory Response

This hypothesis was originally proposed in 2004 by Trayhurn and Wood [1] and discussed in recent articles [131–134]. The best evidence for the “hypoxia hypothesis” is the evidence that adipose tissue is poorly oxygenated in the obese [134, 135]. The mechanisms involved are not understood beyond the accepted paradigm that HIF1*α* activation occurs resulting in activation of NF*κ*B leading to increased gene transcription of inflammatory adipokines. Yin et al. [133] recently suggested that hypoxia in adipose tissue activates lipolysis and inhibits fatty acid uptake by adipocytes leading to activation of an inflammatory response via TLR-4. There is no evidence that activation of lipolysis per se induces an inflammatory response in human fat. Fain et al. [136] reported that growth hormone in the presence of dexamethasone, but not in its absence, stimulated lipolysis by explants of human omental adipose tissue over a 48 hours incubation but this was not accompanied by an increase in IL-8 gene expression or release. 

 Another problem is that while there is evidence that the adipose tissue from the ob/ob mouse is hypoxic in comparison to fat from obese mice, there was no increase in expression of VEGF while there was of hypoxia response genes such as HIF-1*α*, IL-6, Il-1*β*, and TNF*α* [134]. A similar finding has been reported by Halberg et al. [137] and remains to be explained since the current paradigm is that hypoxic tissues release VEGF that leads to increased tissue vascularization. However, the hypothesis may be incorrect or angiogenesis may also require other, as yet unknown, factors. 

An attractive hypothesis is that as fat cells expand there is insufficient neovascularization to keep the cells from becoming hypoxic. This results in activation of HIF1*α* and a variety of responses including increased formation of inflammatory adipokines as well as activation of collagen synthesis and crosslinking of collagen involving lysyl oxidase [137]. There is global upregulation of extracellular matrix formation that hampers oxygen access to the cells and the increased stress resulting from expansion of the fat cells resulted in rupture of very large cells [137]. The fatty acids resulting from breakdown of triacylglycerols released by ruptured fat cells could activate macrophages as well as intact fat cells. 

Alternatively, hypoxia leads to the death of large fat cells and macrophages are drawn to areas of recent cell death by mediators still to be described that are released after cell death, as suggested by Rausch et al. [135]. It may well be that visceral omental fat cells are more liable to lysis which explains why these fat cells are smaller than those found in subcutaneous adipose tissue. Furthermore it is commonly accepted, but may be an over-simplification, that visceral adipose tissue has more macrophages than subcutaneous adipose tissue and releases more inflammatory adipokines. Explants, but not isolated fat cells, of omental adipose tissue have been shown to release more PGE_2_, PAI-1, IL-6, and VEGF than abdominal subcutaneous adipose tissue on a per g basis [62]. Similar results have been reported for IL-8 content of and release by visceral omental as compared to subcutaneous human adipose tissue [138].

## 11. Summary

**Figure 5:**
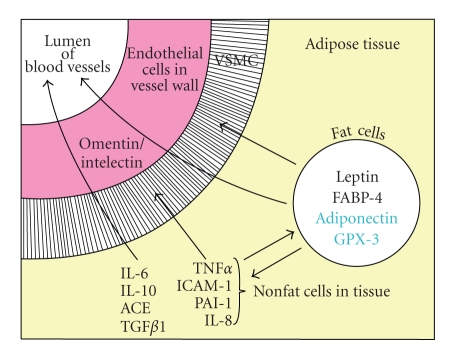
The relationship between adipokine release and paracrine signaling in human adipose tissue. The adipokines are divided into those released by fat cells [leptin, FABP-4, adiponectin, and GPX-3] and those by nonfat cells in adipose tissue [IL-6, IL-8, IL-10, ACE, PAI-1, ICAM-1, TNF*α*, TGF*β*1, and omentin/intelectin]. Adipokines shown in black are those whose circulating levels are elevated in obesity as well as their release by incubated human adipose tissue explants. Circulating levels of adiponectin and GPX-3 are shown in blue since they are not elevated in obesity. Omentin/intelectin is shown as being secreted by the endothelial cells of the blood vessels of omental but not subcutaneous fat [108]. The arrows depict possible targets of the adipokines as the other cells in adipose tissue, as well as vascular smooth muscle cells (VSMC) and endothelial cells in the blood vessel walls plus release into the circulation (lumen of blood vessel).

The data in Figure 5 summarizes the relative release of selected adipokines by fat cells and nonfat cells of human adipose tissue. Of the adipokines shown in the figure only leptin, FABP-4, GPX-3, and adiponectin are expressed at 5 to 80-fold higher levels in fat cells than the other cells present in human fat and primarily released by fat cells. Adiponectin and GPX-3 are listed in blue because their circulating levels are lower in obesity. 

The adipokines with black lettering are those whose circulating levels are enhanced in obesity and whose total release by adipose tissue explants is enhanced in obesity: IL-6, IL-10, ACE, TGF*β*1, ICAM-1, TNF*α*, IL-1*β*, PAI-1, and IL-8 that are released by nonfat cells. However IL-10 may be an anti-inflammatory adipokine primarily released by the nonfat cells, whose circulating levels as well as in vitro release are elevated in obesity. The release of leptin and FABP-4 by fat cells is also enhanced in human obesity. It should be understood that most of these adipokines act locally and whether the changes in circulating levels of adipokines seen in obesity reflect release by adipose or other tissues remains to be established. 

Omentin/intelectin is a novel adipokine preferentially found in visceral fat depots, especially human epicardial fat whose site of origin is the endothelial cells of blood vessels. For this reason it is listed in Figure 5 as being derived from the endothelial cells in the vessel wall. In conclusion, most of adipokines whose circulating levels are elevated in obesity and whose release by human adipose tissue is enhanced in obesity are inflammatory adipokines primarily derived from the nonfat cells of human adipose and other tissues.

## Figures and Tables

**Table 1:** Comparison of release of 37 adipokines by fat cells as compared to the other cells in human adipose tissue ranked by fat cell release along with the effect of obesity on their circulating levels in humans.

Adipokine	Release by nonfatcells in pmoles/g	Release by fatcells in pmoles/g	Effect of obesity on circulating levels
**FABP-4**	**590**	**360**	**Elevated [16–18]**
PGE_2_	1810*	118*	No data
**IL-8**	1120*	87*	**Elevated [21, 22]**
**PAI-1**	78*	18*	**Elevated [19, 23, 24]**
**MCP-1**	74*	9.2*	**Elevated [4, 20, 22, 25]**
**IL-6**	66*	5.1*	**Elevated [4, 25–31]**
**Adipsin**	**26**	**4.1**	**Elevated [28, 32]**
*Adiponectin*	*6*	*4.1*	*Lower* *[25, 33, 34]*
*GPX-3*	*14*	*3.6*	*Lower [35]*
**Leptin**	**0.1**	**1.8**	**Elevated [19, 25, 36]**
**Amyloid A**	**1.3**	**1.6**	**Elevated [19, 32, 37]**
**Migration inhibitor factor **	**2.8**	**1.0**	**Elevated [38]**
Visfatin/PBEF/Nampt	1.0	0.60	No change [39–41]
CD14	1.6	0.56	No change [42]
ZAG	0.7	0.44	No change [17]
Lipocalin-2	5.4	0.27	No change [42, 43]
**Cathepsin S**	**4.4**	**0.26**	**Elevated [44]**
RANTES	2.1	0.21	No change [42] but see [25]
**IL-1Ra**	**4.1**	**0.14**	**Elevated [36]**
Osteoprotegerin	0.1	0.12	No change [42, 45]
**HGF**	**2.8**	**0.11**	**Elevated [46]**
LPL	0.02	0.080	No change [47]
**Haptoglobin**	**0.08**	**0.060**	**Elevated [48]**
**ICAM-1**	**0.27**	**0.056**	**Elevated [4, 29, 49]**
**ACE**	**0.23**	**0.017**	**Elevated [50]**
**IL-10**	**0.53**	**0.020**	**Elevated [28]**
**VEGF**	**0.30**	**0.020**	**Elevated [51]**
**VCAM-1**	**0.46**	**0.016**	**Elevated [29]**
IL-1*β*	0.23	0.013	No data
**TNF** **α**	**0.22**	**0.012**	**Elevated [5, 27, 29–31, 52]**
**TGF-** **β** **1**	**0.17**	**0.009**	**Elevated [53]**
**sTNF RII**	**0.44**	**0.007**	**Elevated [5, 18, 19, 30, 54]**
**NGF**	**0.006**	**0.005**	**Elevated [55]**
VEGFR/sFLT1	0.018	0.002	No change [34]
Resistin	1.8	<0.04	No change [56, 57]
**C** **R** **P**	0.01	<**0.002**	**Elevated [17, 19, 20, 28, 30, 31, 49, 58]**
**I** **L**-18	0.01	<**0.002**	**Elevated [18, 59, 60]**

Those in “bold” are adipokines whose circulating levels have been reported to be elevated in obesity, “italic” those where the circulating levels are lower in obesity, and “normal text” where there is either no effect of obesity or published data. The references are to the reports on circulating levels. The asterisks indicate that the release of these adipokines was almost certainly upregulated over 48 hours. The rate of release for IL-8 over 48 hours extrapolated from release over the first 40 minutes of incubation were 2.2% of those based on the 48 hours release values [61]. The release values for nonfat and fat cells over 48 hours are the averages of subcutaneous and omental adipose tissue from 8 to 12 humans with a BMI of 32 and an equal number with a BMI of 45. These data are from Fain et al., [62] except for haptoglobin [63], resistin [64], MCP-1 [65], TGF*β*1 [66], MIF, Cathepsin S, NGF, IL-1Ra, IL-18 [67], VCAM-1, ACE, adipsin, sTNFR2 [68], CD14, LPL, OPG, Amyloid A, ZAG, GPX-3, FABP-4, ICAM-1, RANTES, visfatin, lipocalin-1 [42] while CRP and VEGFR/sFLT1 are from unpublished experiments.

**Table 2:** Comparison of 100 mRNAs in fat cells as compared to the nonfat cells derived from human omental adipose tissue.

	Ratio of mRNA in fat cells to nonfat cells	Cp value in nonfat cells
mRNAs significantly enriched in fat cells		

**Perilipin**	**128**	**29.3**
**Hormone sensitive lipase [HSL]**	**104**	**32.4**
**Lipoprotein lipase [LPL]**	**79**	**26.5**
**Adiponectin**	**42**	**28.1**
**Retinol binding protein 4 [RBP-4]**	**42**	**29.4**
**Adipose tissue triglyceride lipase [ATGL]**	**37**	**35.4**
**Amyloid protein A1**	**34**	**27.3**
**Leptin**	**28**	**29.2**
**FAT/CD36**	**26**	**25.8**
**11** **β** **-hydroxysteroid dehydrogenase 1 tv1 [11** **β** ** HSD-1]**	**18**	**30.8**
**PPAR** **γ**	**15**	**30.4**
**Uncoupling protein 2 [UCP-2]**	**14**	**28.9**
**Fat specific protein 27/CIDEC**	**13**	**26.0**
**CIDEA**	**12**	**27.0**
**Glutathione peroxidase 3 [GPX-3]**	**9**	**27.0**
**Adipsin/complement D**	**9**	**27.9**
**Zinc ** **α**_2_ **-glycoprotein [ZAG]**	**8**	**28.8**
**Cyclic AMP phosphodiesterase 3B**	**7**	**27.6**
**Angiotensinogen**	**5**	**34.1**
**Toll-like receptor 4 [TLR-4]**	**5**	**34.8**
**Fatty acid binding protein 4 [FABP-4]**	**5**	**20.2**
**Cytochrome c oxidase**	**4**	**28.1**
**Glycerol channel aquaporin 7 [AQP-7]**	**4**	**27.4**
**Adrenomedullin**	**4**	**26.4**
**Akt2/protein kinase B2**	**4**	**27.2**
**Uncoupling protein 1 [UCP-1]**	**4**	**32.6**
**NADPH:quinone oxidoreductase l [NQO-1]**	**3**	**27.2**
**Insulin receptor tv1 [INSR]**	**3**	**27.5**
**GAPDH**	**3**	**26.7**
**CGI-58/ABHD5**	**3**	**26.2**

mRNAs present in both nonfat cells and fat cells		

*Gi* *α* *2 guanine nucleotide binding protein*	*2.1*	*32.0*
*Osteoprotegerin [OPG]*	*1.9*	*31.0 *
*Thrombospondin 1*	*1.8*	*24.5*
*Sodium hydrogen exchanger 1*	*1.6*	*28.9*
*AMPK * *α* *2 catalytic subunit*	*1.5*	*32.9*
*Akt/1protein kinase B1*	*1.5*	*27.4*
*Lipin-1*	*1.5*	*26.5*
*Lipin-2*	*1.5*	*27.4*
*Cyclophilin A*	*1.4*	*29.0*
*Caveolin-1*	*1.4*	*26.6*
*MAP3K8/COT1*	*1.3*	*26.5*
*Receptor interacting protein 140 [RIP 140]*	*1.3*	*24.6*
*Haptoglobin*	*1.0*	*31.2*
*SIRT1/sirtuin1*	*1.0*	*27.1*
*CD14 tv1*	*1.0*	*26.0*
*IL-1 Ra*	*1.0*	*33.3*
*Leucine-rich protein PPR [LRP130]*	*0.87*	*27.0*
*NFKB_1_ [p50]*	*0.81*	*33.0*
*β* *2 adrenergic receptor*	*0.76*	*25.8*
*Hypoxia inducible factor 1* *α* * [HIF-1* *α* *]*	*0.71*	*27.4*
*CD 68*	*0.71*	*24.0*
*RAB6*	*0.66*	*25.0*
*Peroxisome proliferator activator receptor-* *γ* * coactivator 1* *α* * [PGC-1] *	* 0.57*	* 30.0*
*Apelin*	*0.57*	*33.1*
*MAP4K4 tv 2*	*0.57*	*29.2*
*Heme oxygenase-1 [HMOX-1]*	*0.57*	*24.4*
*Renin receptor*	*0.54*	*25.8*
*β* *1 adrenergic receptor*	*0.50*	*27.8*
*Resistin*	*0.33*	*33.6*

mRNAs significantly enriched in nonfat cells		

Endothelial nitric oxide synthase [eNOS]	0.44	30.2
PI-3 kinase catalytic subunit	0.38	27.2
Cathepsin S	0.38	31.4
NFKB p65	0.35	29.2
Mitochondrial superoxide dismutase-2 tv1 [SOD2]	0.35	21.0
Tumor necrosis factor-*α* receptor 2 [TNFR-2]	0.29	31.2
Nerve growth factor beta polypeptide [NGF]	0.29	31.5
Interleukin 10 [IL-10]	0.25	30.0
Visfatin/PBEF/Nampt	0.25	21.9
PR domain containing 16 [PRDM16] tv1	0.25	31.5
Tumor necrosis factor *α* [TNF*α*]	0.25	30.5
Glycogen synthase kinase 3*β*	0.23	32.0
Interleukin 8 [IL-8]	0.20	23.6
Osteocalcin	0.20	30.2
*α*1 glycoprotein	0.20	33.8
Complement C-3	0.19	25.9
Interleukin 1*β* [IL-1*β*]	0.19	25.7
Prostaglandin D_2_ synthase [PGDS]	0.18	23.9
Tribbles 3 [TRB3]	0.18	30.6
Plasminogen activator inhibitor 1 [PAI-1]	0.18	27.3
Bone morphogenetic protein 7 [BMP-7]	0.16	31.8
Intercellular adhesion molecule 1 [ICAM-1]	0.16	25.2
Cyclooxygenase 2 [COX-2]	0.15	27.4
RANTES	0.15	30.0
Angiotensin 1 converting enzyme [ACE]	0.15	31.0
Interleukin 6 [IL-6]	0.14	25.0
Endocannabinoid receptor 1	0.14	25.8
Collagen type VI/PBEF1*α*3 tv1 [COL6-A3]	0.14	29.9
NADPH oxidase p67^phox^	0.13	30.5
TGF*β*-1	0.12	27.3
NADPH oxidase p47^phox^	0.12	32.4
Lipocalin 2	0.12	33.5
Butyrylcholinesterase	0.11	33.2
Angiotensin II receptor-1 tv1 [AT_1_R]	0.10	25.7
Omentin/intelectin	0.09	25.0
Monocyte chemoattractant protein 1 [MCP-1]	0.07	23.2
VEGF receptor [VEGFR/FLT1]	0.07	27.8
25-Hydoxyvitamin D3 1*α* hydroxylase [25D3-1*α*]	0.06	26.8
Endothelin-1	0.06	27.8
Angiotensin II receptor-2 [AT_2_R]	0.06	33.1
Vaspin	0.06	28.1

Those in “bold” are the mRNAs significantly enriched in fat cells, “italic” those mRNAs present in both nonfat cells and fat cells to the same extent, and “normal text” those mRNAs significantly enriched in nonfat cells. The data are based on quantitative PCR analysis of mRNA expression [14, 82, 83]. The ratios were derived from the log_2 _ΔΔCp of the ΔCp (crossing point) for each mRNA, except cyclophilin, of the nonfat cells (pooled undigested tissue + SV fractions) obtained by collagenase digestion of human omental adipose tissue subtracted from the ΔCp values of fat cells isolated from the same tissue. The ratio for cyclophilin is based on log_2_ of the ΔCp values for cyclophilin. A ratio above 1 means that the amount of mRNA is greater in the fat cells than in the nonfat cells. The values are shown as the means ± SEM of 4 to 21 paired experiments comparing nonfat cells to fat cells derived from the same individual. Tv1 or 2 stands for transcript variant 1 or 2. The data are from Fain et al. [82, 83] or from unpublished data.

**Table 3:** Correlation between total release by explants of human fat and total fat mass as well as the change in mRNA over 48 hours incubation.

Adipokine	*r* value for correlation of release with fat mass	Change in mRNA over 48 hour (ratio)
Those with significant positive correlations of total release with fat mass		

**IL-8**	**0.85**	**510**
Lactate	0.81	
FABP-4	0.73	0.06
**IL-10**	**0.70**	**13**
**TGF** **β** **1**	**0.69**	**8**
**Visfatin**	**0.67**	**30**
**IL-1** **β**	**0.65**	**120**
**IL-6**	**0.65**	**675**
CD14	0.64	1.7
Endothelin-1	0.63	1.4
**sICAM-1**	**0.61**	**6.1**
TNF*α*	0.59	
GPX-3	0.56	0.33
**Lipocalin-2**	**0.54**	**34**
**PAI-1**	**0.53**	**295**
ACE	0.52	0.55
LPL	0.51	0.14

Those with no significant correlations of total release with fat mass		

Amyloid A	0.18	0.50
**MCP-1**	**0.12**	**37**
**IL-1 Ra**	**0.10**	**9**
Adipsin	0.01	0.23
**Osteoprotegerin**	**0.01**	**4.9**
VEGF	−0.04	
RANTES	−0.05	1.2
Cathepsin S	−0.07	
ZAG	−0.09	0.25
VCAM-1	−0.30	
**NGF** **β**	−**0.30**	**13**

The values shown in bold are for adipokines whose gene expression was upregulated over the 48 hours incubation. The changes in mRNA as measured by qRTPCR over 48 hours were based on comparison of unincubated adipose tissue explants with those after 48 hours and shown as the ratios derived from the ΔCp values [95]. The Pearson correlation coefficient (*r*) was derived by plotting the total release over 48 hours by the average of values for omental and subcutaneous adipose tissue versus the calculated total fat mass in 20–22 obese women as described in Fain et al. [66–68]. The correlation coefficients have been published for TGF*β*1 [66], cathepsin S, nerve growth factor *β* (NGF*β*), interleukin-1 receptor antagonist (IL-1Ra) and interleukin 18 (IL-18) [67] as well as those for adipsin, vascular cell adhesion molecule 1 (VCAM-1), and angiotensin1 converting enzyme (ACE) [68]. The correlation coefficients for the following are derived from Fain et al. [42]: endothelin-1, zinc-*α*2-glycoprotein (ZAG), lipocalin-2, CD14, RANTES, lipoprotein lipase (LPL), osteoprotegerin, fatty acid binding protein 4 (FABP-4), visfatin, glutathione peroxidase-3 [GPX-3], intracellular cell adhesion molecule 1 [ICAM-1], and amyloid A while those for IL-8, IL-6, PAI-1, TNF*α*, IL-10, VEGF, and IL-1*β* were derived from Fain et al. [62]. The changes in mRNA over 48 hours are from Fain et al. [95].

**Table 4:** Comparison of mRNAs in human mesenteric and subcutaneous as compared to omental adipose tissue from extremely obese women.

mRNA	subcutaneous as ratio of omental	mesenteric as ratio of omental
mRNAs lower in both subcutaneous and mesenteric as compared to omental		

Omentin/intelectin	0.01 ± 0.01***	0.14 ± 0.03***
Angiotensin II receptor 2 [ATR_2_]	0.04 ± 0.01***	0.35 ± 0.10**
Haptoglobin	0.08 ± 0.01***	0.38 ± 0.10***
Nerve growth factor *β* [NGF*β*]	0.22 ± 0.02***	0.44 ± 0.14**
Complement factor C3	0.31 ± 0.05***	0.47 ± 0.08***
VEGFR/FLT-1	0.41 ± 0.09***	0.66 ± 0.15*
PGC-1*α*	0.41 ± 0.14***	0.73 ± 0.06**
**Insulin receptor**	0.47 ± 0.08***	0.44 ± 0.14*
SIRT1/sirtuin 1	0.47 ± 0.15**	0.44 ± 0.09***
Collagen VI *α*3	0.48 ± 0.16**	0.60 ± 0.11*

mRNAs lower or higher in subcutaneous but not in mesenteric as compared to omental		

**Uncoupling protein 1 [UCP-1]**	0.07 ± 0.01***	**1.23 ± 0.60**
Prostaglandin D_2_ synthase	0.27 ± 0.05***	1.06 ± 0.12
**Angiotensinogen**	0.33 ± 0.04***	**0.87 ± 0.20**
Bone morphogenetic protein 7 [BMP-7]	0.35 ± 0.19**	1.02 ± 0.16
**Zinc *α*2 glycoprotein [ZAG]**	0.44 ± 0.11***	**0.83 ± 0.06**
NF*κ*B1 [p50]	0.54 ± 0.15*	0.81 ± 0.19
**Cytochrome C oxidase**	1.45 ± 0.13***	**1.10 ± 0.13**
Angiotensin II receptor 1 [ATR_1_]	1.62 ± 0.18***	0.93 ± 0.21
NAPDH oxidase [p67^phox^ ]	1.90 ± 0.22***	1.10 ± 0.17
CD 14	2.30 ± 0.37***	0.85 ± 0.25
25-hydroxyvitamin D3 1*α* hydroxylase	3.00 ± 0.64**	0.90 ± 0.48
**Retinol binding protein 4 [RBP-4]**	3.10 ± 0.26***	**0.87 ± 0.20**
Interleukin 6 [IL-6]	3.50 ± 0.55***	0.66 ± 0.34
Osteopontin	4.90 ± 0.51***	1.07 ± 0.21

mRNAs lower or higher in mesenteric but not in subcutaneous as compared to omental		

Monocyte chemoattractant protein 1	1.15 ± 0.45	0.15 ± 0.04**
Interleukin 1*β* [IL-1*β*]	0.62 ± 0.30	0.20 ± 0.04***
**Adrenomedullin**	**0.97 ± 0.09**	0.38 ± 0.13*
**PPAR** **γ**	**1.23 ± 0.15**	**0.44 ± 0.11** **
*β*1 adrenergic receptor	1.00 ± 0.07	1.74 ± 0.27*

mRNAs higher in subcutaneous and lower in mesenteric as compared to omental		

Plasminogen activator inhibitor 1	1.62 ± 0.27*	0.20 ± 0.04***

mRNAs higher in subcutaneous and mesenteric as compared to omental		

*α*1 glycoprotein	7.00 ± 0.86***	1.74 ± 0. 27**

mRNAs the same in subcutaneous and mesenteric as compared to omental (ratios were 0.50 to 1.50 of that in omental and not statistically significant).

ACE, **adiponectin, adipsin, amyloid A**, cathepsin S, caveolin-1, **CIDEA,** CD68, cyclophilin, endothelin-1,** FABP-4, FAT/CD36, **Gi*α*2, **GPX-3, **heme oxygenase-1, HIF1*α*, **11** **β** **HSD-1, HSL,** IL-8,** leptin, **lipocalin-2**, LPL**, NADPH oxidase [gp91^phox^], NGF*β* [p65 RelA], eNOS, osteoprotegerin, **perilipin,** PRDM-16, TNF*α*, **Toll like receptor 4, UCP-2, ** visfatin.

The values were obtained by qPCR as described in [106] and are expressed as the ratio ± sem of 5 to 15 paired comparisons from as many different individuals of the amount of mRNA in mesenteric and subcutaneous fat as compared to omental fat from the same woman. The mRNAs enriched in fat cells by at least 3-fold are shown in bold. Statistically significant differences are denoted as follows:  *P ≥ .05, **P ≥ .01, and ***P ≥ .005.
